# Lactobacillus Proteins Are Associated with the Bactericidal Activity against *E. coli* of Female Genital Tract Secretions

**DOI:** 10.1371/journal.pone.0049506

**Published:** 2012-11-19

**Authors:** Sabah Kalyoussef, Edward Nieves, Ellen Dinerman, Colleen Carpenter, Viswanathan Shankar, Jamie Oh, Berta Burd, Ruth H. Angeletti, Karen W. Buckheit, David N. Fredricks, Rebecca P. Madan, Marla J. Keller, Betsy C. Herold

**Affiliations:** 1 Department of Pediatrics, Albert Einstein College of Medicine, Bronx, New York, United States of America; 2 Department of Developmental and Molecular Biology, Albert Einstein College of Medicine, Bronx, New York, United States of America; 3 Department of Biochemistry, Albert Einstein College of Medicine, Bronx, New York, United States of America; 4 Department of Epidemiology and Population Health, Albert Einstein College of Medicine, Bronx, New York, United States of America; 5 Department of Medicine, Albert Einstein College of Medicine, Bronx, New York, United States of America; 6 Department of Microbiology-Immunology, Albert Einstein College of Medicine, Bronx, New York, United States of America; 7 ImQuest Biosciences, Fredrick, Maryland, United States of America; 8 Department of Medicine, University of Washington, Seattle, Washington, United States of America; Columbia University, United States of America

## Abstract

**Background:**

Female genital tract secretions are bactericidal for *Escherichia (E.) coli ex vivo*. However, the intersubject variability and molecules that contribute to this activity have not been defined.

**Methods:**

The bactericidal activity and concentration of immune mediators in cervicovaginal lavage (CVL) collected from 99 healthy women were determined.

**Results:**

CVL reduced the number of *E. coli* colonies by 68% [−26, 100] (median [range]). CVL were active against laboratory and clinical isolates of *E. coli,* but were inactive against Lactobacillus species. Bactericidal activity correlated with the concentration of protein recovered (p<0.001), but not with cytokines, chemokines or antimicrobial peptides. Four CVL with>90% inhibitory activity (active) and two with<30% activity were subjected to MS/MS proteomic analysis. 215 proteins were identified and six were found exclusively in active samples. Four of these corresponded to *Lactobacillus crispatu*s or *jensenii* proteins. Moreover, culture supernatants from *Lactobacillus jensenii* were bactericidal for *E. coli.*

**Conclusion:**

Both host and commensal microbiota proteins contribute to mucosal defense. Identification of these proteins will facilitate the development of strategies to maintain a healthy vaginal microbiome and prevent colonization with pathogenic bacteria such as *E. coli* that increase the risk for urinary tract infections, preterm labor and perinatal infection.

## Introduction

*Escherichia coli (E. coli),* which are the predominant facultative anaerobes of the gastrointestinal tract, are the major cause of urinary tract infections (UTIs), the second most frequently isolated pathogen in neonatal sepsis, and the most common pathogen isolated from very low birth weight preterm infants [1]. Colonization of the vagina by uropathogens may be a critical event preceding UTIs and may contribute to preterm birth [2]. In the Vaginal Infections and Prematurity Study, which involved over 13,000 pregnant women, an increase in *E. coli* or *Klebsiella pneumoniae* in vaginal cultures at delivery was associated with an increased risk of preterm birth and a higher adjusted odds ratio for preterm birth than any other factor [3].

Precisely what regulates *E. coli* vaginal colonization is not well understood. Mucosal defenses include an intact epithelial barrier, acidic pH provided by lactic acid and hydrogen peroxide-producing flora, and antimicrobial molecules secreted by epithelial and immune cells. The importance of soluble factors in preventing *E. coli* colonization is supported by observations that genital tract secretions collected by cervicovaginal lavage (CVL) or swab are bactericidal for *E. coli* [4]–[6]. The most well characterized mucosal antimicrobial peptides are defensins. Early studies showed that human neutrophil peptides 1–3 (HNP1-3), α-defensins secreted primarily by neutrophils, were bactericidal for several pathogenic bacteria including *E. coli,* and mechanistic studies demonstrated that HNP1-3 sequentially permeabilized the outer and inner membrane of *E. coli* [7]. In a small study, vaginal secretions from women with bacterial vaginosis (BV) were found to have lower concentrations of HNP1-3 (and other antimicrobial peptides) and little or no anti-*E. coli* activity compared to healthy controls; the activity was partially restored following treatment with metronidazole [5].

**Figure 1:**
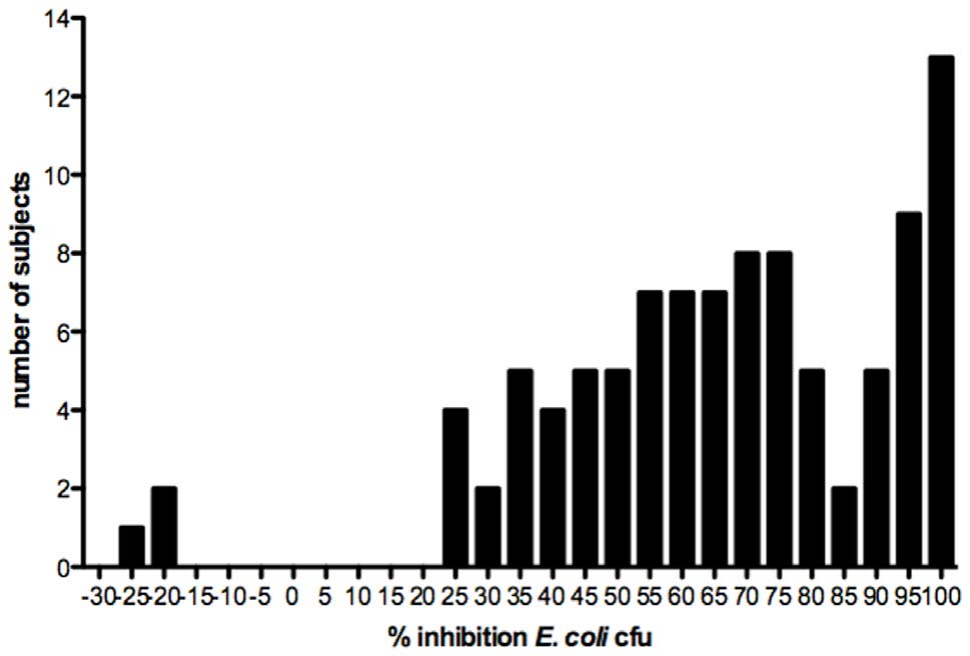
Distribution of CVL *E. coli* bactericidal activity from a cohort of 99 healthy females. Each CVL or control fluid (200 µg/mL of bovine serum albumin in normal saline) was mixed with ∼10^9^ cfu of *E. coli* for 2 h. The mixture was then diluted to yield 800–1000 cfu on control plates and plated in duplicate on tryptic soy agar plates. Colonies were counted after 24 h incubation and the mean percentage inhibition relative to the control plates was determined.

Building from this framework, the current study was designed to further characterize the endogenous *E. coli* activity in healthy, non-pregnant women and to apply proteomic approaches to identify proteins that may contribute to host defense. Identification of the molecules that contribute to the antimicrobial activity will accelerate the development of strategies to boost host defense and the identification of biomarkers that predict a greater risk for vaginal colonization with pathogenic bacteria associated with recurrent UTIs, preterm labor, and perinatal sepsis.

## Methods

### Ethics Statement

The Albert Einstein College of Medicine Institutional Review Board approved the studies and all subjects provided written informed consent.

**Figure 2:**
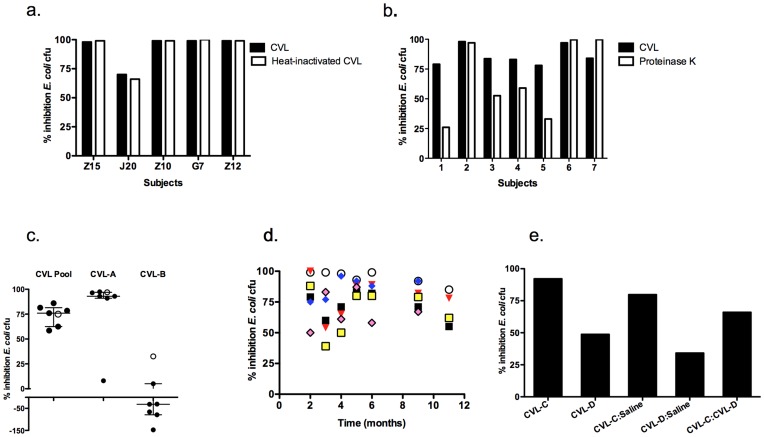
Stability of bactericidal activity. (a) Five lavage samples were tested for bactericidal activity before and after heat inactivation at 56°C for 30 minutes. Each bar is the mean percentage inhibition of colonies formed on duplicate plates; error bars are too small to be visualized. (b). Bactericidal activity was tested in seven CVL samples treated with or without 100 µg/mL of proteinase K. Each bar is the mean percentage inhibition of colonies formed on duplicate plates relative to control plates (bacteria treated with control buffer). (c) The bactericidal activity of a pool of five individual CVL samples and a randomly selected single CVL with high activity (CVL-A) and one with low activity (CVL-B) were tested against the lab strain of *E. coli* (open circles) or six clinical isolates. Each symbol represents mean percentage inhibition in cfu from duplicate plates relative to the control plates and the bars denote mean (sd). (d) Stored aliquots of CVL obtained from six subjects (each indicated by a different color symbol) were tested at the indicated intervals from time of collection up to one-year post collection. Each symbol represents the mean percent inhibition of *E. coli* cfu obtained from duplicate plates at the indicated times. (e) CVL from a subject with high activity (CVL-C) and less activity (CVL-D) were mixed 1∶1 with saline or with each other and then tested for ability to inhibit *E. coli*. Results are presented as percentage inhibition relative to control plates and are the means obtained from two independent experiments.

### Participants

CVL samples were obtained from healthy women who enrolled in microbicide safety studies. Ninety-nine women who were between the ages of 18 and 50 years were recruited between February 2008 and November 2010. Subjects were asked to abstain from intercourse for at least 48 h and were excluded for, pregnancy, breastfeeding, menopause, HIV, genitourinary infection, BV (diagnosis by Amsel’s criteria), abnormal Pap test, positive semen test, and oral or topical antibiotic use. At the study visit, a brief demographic, medical and sexual history was obtained. Subjects had a urinalysis and culture, pregnancy test, Pap test (if not performed within past 12 months) and gynecological examination for detection of BV, *Trichomonas vaginalis*, *Candida* species and semen. *Neisseria gonorrhoeae* and *Chlamydia trachomatis* infection were determined by nucleic acid amplification testing. Blood was drawn for HIV-1 (ELISA), syphilis (RPR), and type-specific HSV antibody testing (HerpeSelect, Focus Diagnostics, Cypress, CA).

**Table 1:** Distribution of demographic, sexual behaviors and history of STI and bivariate regression estimate for *E. coli* bactericidal activity.

Variable (n from whom data available)	Subcategory	n (%)	β (SE)	p value
Age (99) mean (SD)		30.63 (7.24)	−0.25 (0.38)	0.51
Race (99)	White	24 (24.24)		
	Black	34 (34.34)	−6.43 (7.27)	0.37
	Hispanic	25 (25.25)	−9.20 (7.79)	0.24
	Other	16 (16.16)	−5.89 (8.80)	0.51
Cigarette smoking (99): Yes		20 (20.20)	−8.95 (6.74)	0.18
Current number of sex partners (99)	0	31 (31.31)		
	≥1	68 (68.69)	6.37 (5.85)	0.28
Number of sex partners in last year (median, range) (68): (Yes)		1 (0, 6)	6.74# (10.97)	0.54
History of vaginal intercourse (99): Yes		95(95.96)	18.39 (13.75)	0.18
History of anal intercourse (85): (Yes)		34 (40.0)	4.38 (6.11)	0.47
History of douching (99): (Yes)		24 (24.24)	−2.94 (6.37)	0.64
History of urinary tract infection (94): (Yes)		32 (34.04)	−2.09 (5.89)	0.72
History of tampon use (94): (Yes)		64 (64.65)	10.43 (5.62)	0.07
Current form of contraception: (Yes)	Hormonal (99)	26 (26.26)	9.79 (6.13)	0.11
	IUD (99)	8 (8.08)	4.41 (10.02)	0.66
	Tubal ligation (99)	6 (6.06)	−12.97(11.38)	0.26
	Male condom (85)	39 (45.88)	−3.06 (6.01)	0.61
	Female condom (85)	8 (9.41)	−27.61 (9.82)	0.01
	Withdrawl (85)	7 (8.24)	8.55 (10.88)	0.43
History of genital tract infection (Yes) (99)	Chlamydia	17 (17.17)	0.77 (7.25)	0.95
	Gonorrhea	5 (5.05)	−11.35 (12.43)	0.36
	Trichononas	9 (9.09)	1.50 (9.5)	0.87
	Bacterial vaginosis	18 (18.18)	−6.79 (7.05)	0.34
	Candida	56 (56.57)	4.24 (5.49)	0.44
	Genital herpes	2 (2.02)	−1.22 (19.43)	0.95
	Genital warts	4 (4.04	5.39 (13.87)	0.69
HSV serostatus (77)	HSV-1+	45 (58.44)	−6.4 (6.57)	0.33
	HSV-2+	17 (22.08)	1.19 (7.86)	0.87

# modeled as dichotomized variable ≥1 partner(s) vs. no partners.

Vaginal pH was measured from a swab of the lateral vaginal wall (Whatman pH paper, pH 3.8–5.5). CVL was performed by washing the cervix and posterior fornix with 10 ml of normal saline (pH∼5.0). The samples were transported on ice to the laboratory, centrifuged at 700 g × 10 minutes and the supernatants divided into aliquots and stored at −80°C.

### Antibacterial Assay

*E. coli* (ATCC strain 4382627) was grown overnight to stationary phase and then 3 µl of bacteria (∼10^9^ colony forming units (cfu)/ml) were mixed with 27 µl of CVL or control buffer (20 mmol/L potassium phosphate, 60 mmol/L sodium chloride, 0.2 mg/ml albumin, pH 4.5) and incubated at 37°C for 2 hours [8]. The mixtures were then further diluted in buffer (to yield 800–1000 colonies on control plates) and plated on agar enriched with trypticase soy broth. Colonies were counted using ImageQuant TL v2005 after an overnight incubation at 37°C. All samples were tested in duplicate and the percentage inhibition was determined relative to the colonies formed on the control plates. In pilot studies, 10 random CVL supernatants were directly plated onto agar with enriched trypticase soy broth and incubated overnight at 37°; none yielded bacteria.

**Table 2:** Outcome variables including bactericidal activity, vaginal wall pH, and concentration of immune mediators and bivariate regression estimate for *E. coli* inhibition.

Variable (number from whom data available)	median (range)	β (SE)*	p value
*E. coli* inhibition (%) (99)	68 (−26, 100)		
Vaginal wall pH (95)	4.5 (3.5, 5.5)	−4.14 (5.89)	0.48
Protein µg/ml (95)	302 (0.5, 1059)	0.06 (0.01)	<0.0001
IL-1α pg/ml (59)	45.2 (1.6, 1481.4)	8.07 (6.74)	0.23
IL-1β pg/ml (59)	3.0 (0.2, 468)	0.20 (4.22)	0.96
MIP-1α pg/ml (59)	9.9 (1.1, 177.17)	−1.15 (7.97)	0.88
MIP-1β pg/ml (59)	10.0 (0.6, 138.8)	−3.82 (6.90)	0.58
RANTES pg/ml (59)	2.8 (0.5, 24.8)	8.52 (8.50)	0.32
IL-8 pg/ml (59)	401.39 (9.94, 16995.7)	1.06 (5.54)	0.84
IL-6 pg/ml (59)	12.5 (0.2, 101.06)	−5.56 (5.82)	0.37
IL-1ra pg/ml (59)	7831 (662, 17684.01)	−22.07(12.96)	0.09
Interferon α pg/ml (59)	12.25# (12.2, 71.8)	−6.88 (11.51)	0.55
Interferon γ pg/ml (59)	1.16# (0.05, 11.04)	−13.04 (11.42)	0.26
SLPI ng/ml (65)	247.28 (0.1, 10117)	2.75 (5.16)	0.60
HNP1-3 ng/ml (59)	39.5 (2., 1398.8)	2.73 (4.96)	0.58
Lysozyme ng/ml (59)	150 (12.5, 6326.6)	5.68 (6.57)	0.39
Lactoferrin ng/ml (57)	481(11,15929)	−6.22 (5.57)	0.27
IgA ng/ml (56)	1103.5 (15, 7519)	11.09 (7.20)	0.13
IgG ng/ml (58)	7021 (295, 300095)	3.08 (7.25)	0.67

* β coefficients and p values for immune mediators refer to log transformed values.

# Modeled as dichotomized variable: Interferon α (>or≤12.25) and Interferon γ (>or≤0.05).

### Antibacterial Activity of Spent *Lactobacillus Jensenii (L. Jensenii)* Culture Supernatants

*L. jensenii* (ATCC 25258, strain 63G) was grown in de Man, Rogosa and Sharpe (MRS) media (Sigma) overnight at 37°C and 5% CO_2_. Spent culture supernatants (SCS) were prepared by centrifuging overnight cultures of bacteria at 2000 g for 15 min at room temperature and then filtering the supernatant (0.22 µM syringe filter). The bactericidal activity of SCS (pH 4.3) or SCS adjusted to pH 6.3 with sodium hydroxide (to match the pH of LB broth) was determined.

**Figure 3:**
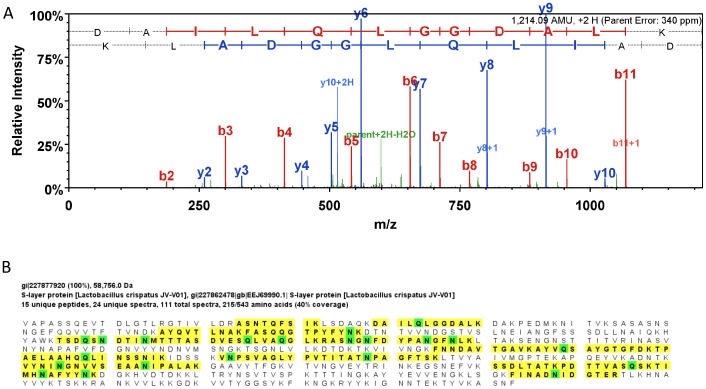
MS/MS data of the S-layer protein of *L. crispatus*. MS/MS of the precursor doubly charged ion at *m/z* 608.05 matching to the S-layer protein peptide DAILQLGGDALK from *L. crispatus* (A). The other matching peptides are highlighted in yellow and the green highlights are residues that have undergone deamidation (B).

**Table 3:** Proteins identified in more than one active, but not in the inactive CVL samples by LC-ESI-MS/MS.

Candidate Proteins (Accession Number)	Origin	MW (kD)	1A	1B	1C	2A	2B	2C	3A	3B	3C	4A	4D
S-layer protein gi [227877920]	*L. crispatus*	59	X	X	X	X	X	X				X	X
Bacterial surface layer protein gi [312983924]	*L. crispatus*	47	X	X	X	X	X	X					
Cell separation protein gi [256844356]	*L. crispatus*	56			X	X		X					
Adhesion exoprotein gi [282932703]	*L. jensenii*	65		X		X		X					
Protein-glutamine γ-glutamyltransferase E precursor gi [189458821]	Human	77		X	X	X	X	X					
Bone-derived growth factor gi [1203965]	Human	86		X	X	X	X	X					

Proteomic analysis was performed on the starting material (A), flow through (B), and retentate (C) after fractionation with a Centricon 50 kDa spin column from an active CVL pool (1) or two different individual CVL (2, 3). Sample 4 was separated on a gel filtration column and the starting material (A) and the fraction with the greatest bactericidal activity (D) were evaluated.

### Clinical Isolates

Six clinical isolates of *E. coli* were provided by the Montefiore Medical Microbiology Lab to test for variability of bactericidal activity with diverse strains. Isolates were obtained from blood (isolate 1), urine (isolates 3 and 6), and genital cultures (isolates 2, 4, and 5). Each isolate was grown in 5 mL LB broth overnight and diluted as needed to an optical density ranging from 0.2–0.4 prior to treatment with CVL.

**Figure 4:**
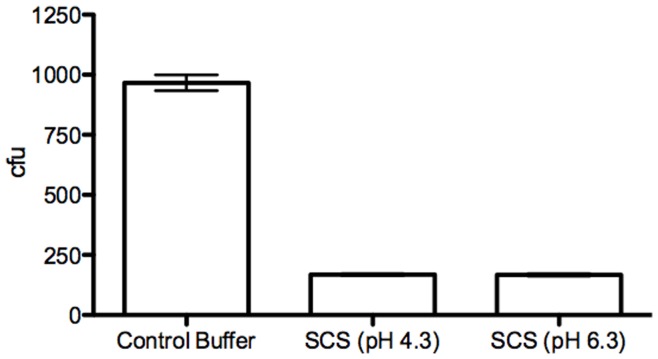
*L. jensenii* spent culture supernatants are bactericidal for *E. coli.* * E. coli* were mixed with control buffer, SCS (pH 4.3) or SCS adjusted to pH 6.3 for 2 hours and then the mixture was diluted and plated in duplicate on tryptic soy agar plates. Colonies were counted after 24 h incubation; results are means (sd) from duplicate plates from a representative experiment.

### Measurement of Immune Mediators

Concentrations of interleukin (IL)-1α, IL-1β, IL-6, IL-8, interferon (IFN)-α, IFN-γ, IL-1 receptor antagonist (IL-1ra), macrophage inflammatory protein (MIP)-1α, MIP-1β, and RANTES (chemokine regulated on activation, normal, T-cell expressed and secreted) were quantified for each CVL sample using a multiplex proteome array with beads from Millipore (Billerica) using Luminex^100^ and analyzed using StarStation (Applied Cytometry Systems). Total protein levels for each CVL sample were determined using a MicroBCA assay (ThermoScientific). Commercial ELISA kits were used to quantify levels of secretory leukocyte inhibitor (SLPI; R&D Systems), HNP1-3 (Hycult Biotechnology, Uden, The Netherlands), lactoferrin (Calbiochem, San Diego, CA), lysozyme (Alpco Diagnostics, Salem, NH), IgG and IgA (Cygnus Technologies, Southport, NC).

### Heat Stability, Susceptibility to Proteinase K, and Proteomic Analyses

CVL aliquots were tested for anti-*E. coli* activity after heating to 56°C for 30 minutes and allowed to cool for 20 minutes before being tested. To determine if the activity mapped to proteinase-sensitive proteins, aliquots were treated with 100 µg/mL of Proteinase K prior to assessment of anti-*E. coli* activity. Mass spectrometry was performed on unfractionated or fractionated CVL samples. Individual or pooled aliquots of CVL were applied to the upper chamber of a Centricon-50 centrifugal filter unit (Amicon Ultra, Cat no UFC805008) and centrifuged at 4000 g for 10 minutes. The flow through and retentate were recovered, adjusted to their original volumes by dilution in normal saline, divided into aliquots and stored at −80°C. The unfractionated and fractionated material from three active (>90%) and two inactive (<30%) CVL underwent LC ESI-MS/MS (liquid chromatography electrospray ionization tandem mass spectrometry) analysis. The samples (0.6–1.0 ml) were initially concentrated using three-layer sandwich gel electrophoresis with 10–12% acrylamide and digested prior to LC ESI-MS/MS [9]. An additional active CVL underwent MS/MS analysis after separation in a gel filtration column (Superdex 200 10/300 GL; GE Healthcare) and in-solution trypsin digestion. DTA files were created from the raw LC-MS/MS data, and searched with Mascot (Version 3.00.05; Matrix Science Inc., Boston, MA) against the NCBI database (May 31, 2011). A false discovery rate for peptide identification was assessed by decoy database searching. The following parameters were used for all searches: trypsin; one missed cleavage; fixed modification of carbamidomethylation (Cys); variable modifications of deamidation (Asn and Gln), pyro-Glu (N-term Gln and Glu) and oxidation (Met); monoisotopic masses; peptide mass tolerance of 3 Da; and product ion mass tolerance of 0.6 Da. Proteins were considered identified if they had at least one bold red peptide (BR; the most logical assignment of a peptide to a protein and prevents duplicate homologous proteins from being reported) and a peptide ion score cut-off of 65 or greater (corresponding to p<0.05). Scaffold was used to validate MS/MS based peptide and protein identifications (version 3.3; Proteome Software, Portland, Oregon, USA).

### Quantification of Vaginal Microbiota

CVL pellets were subjected to DNA extraction as previously described [10]. Quantitative polymerase chain reaction (qPCR) assays utilizing primers and probes specific to each bacterium’s 16S rRNA gene were then used to quantify concentrations of *L. crispatus*, *L. jensenii*, *L. iners* and *Gardnerella (G.) vaginalis* [11]. Quantified bacterial levels were expressed as copies of bacterial DNA per CVL sample.

### Statistical Analysis

Descriptive outcomes are presented as medians (ranges). To examine the relationship between bactericidal activity and immune mediators, the non-normal variables were log transformed and those variables that correlated with bactericidal activity in bivariate analyses with p value ≤0.25 were considered for inclusion as predictors in a multivariable linear regression model. Multivariable linear regression analyses were performed using backward elimination selection procedure. Assumption diagnostics and computation diagnostics were utilized to examine any violation of statistical assumptions and colinearity. All analyses were performed using SAS version 9.2 (SAS Institute, Cary, NC).

## Results

### Bactericidal Activity of CVL

CVL were collected from 99 healthy women who were being screened for participation in vaginal microbicide safety studies. The clinical characteristics are summarized in Table 1. The majority of CVL supernatants exhibited bactericidal activity and reduced the *E. coli* cfu by 68% [−26, 100] (median [range]) (Figure 1). Notably, CVL from three participants increased the number of cfu. To further define the nature of the inhibitory activity, aliquots from five CVL supernatants were tested before and after heat inactivation, which unfolds many protein structures. There was no loss in antimicrobial activity (Fig. 2a). Treatment of other samples with proteinase K, a protease that cleaves at the carboxyl side of aliphatic, aromatic or hydrophobic residues, reduced the antibacterial activity in four of seven CVL tested (Fig. 2b).

To determine if CVL supernatants were active against diverse strains, a pool of CVL supernatants from 5 subjects and a randomly selected single CVL with high activity (CVL-A) and one with low activity (CVL-B) were tested against 6 clinical isolates and the lab strain of *E. coli* in parallel (Fig. 2c). There was little variability in the activity against different strains and the pool and CVL-A reduced the number of cfu by 74±10% and 82±33% (mean ± sd), respectively. CVL-A, however, was inactive against one clinical isolate. In contrast, CVL-B exhibited little inhibitory activity and increased the number of cfu recovered following incubation with 5 of the clinical isolates. None of the CVL were bactericidal against *L. crispatus* (data not shown).

Biological activity may be reduced after prolonged storage, thus limiting its value as a biomarker. To assess this, individual aliquots from 6 subjects with inhibitory activity ranging from 50%–100% were thawed monthly and tested for antibacterial activity. There was little change in bactericidal activity over 11 months, with coefficients of variations between aliquots ranging from 10–26.8% (Fig. 2d.).

### Combining Inactive with Active CVL Restores Anti-*E. coli* Activity

To determine whether inactive CVL samples were deficient in antimicrobial molecules or possessed an inhibitor, we performed mixing studies. CVL-C inhibited *E. coli* by 99% undiluted and by 83% when diluted 1∶1 with saline whereas CVL-D inhibited *E. coli* by 59% and by 37% when diluted 1∶1 with saline. Combining the two yielded a mean *E. coli* inhibition of 77%, indicating restoration in the activity of CVL-D (Fig. 2e). These findings suggest that low activity may reflect the loss of protective molecules rather than the presence of inhibitory factors.

### Correlation of *E. coli* Activity with Mucosal Immune Mediators

The concentrations of protein, and select cytokines, chemokines, and antimicrobial peptides were determined (Table 2). The impact of immune mediators and clinical characteristics on the bactericidal activity was evaluated by bivariate regression estimates (Tables 1 and 2). Only total protein correlated significantly with bactericidal activity. In a multivariate analysis which included race, vaginal pH, history of smoking, total protein, log_10_ IL-1α, log_10_ IL-1ra and log_10_ IgA, protein retained significance (p<0.0001) (note: female condom use and hormonal contraception were not considered in the model because of the small sample size across categories). A 100 µg/ml increase in protein concentration increased the bactericidal activity by 10%, after adjusting for other variables. IL-1ra also retained borderline significance (p = 0.07); 1 log_10_ unit increase in IL1-ra was associated with 22.3% reduction in bactericidal activity.

### Fractionation and Proteomic Studies Identify Proteins Unique to Active CVL

The observation that the bactericidal activity correlated significantly with total protein, but not with any of the immune mediators measured, prompted application of proteomics to identify other candidate mediators of bactericidal activity. Individual CVL or a pool of samples from 5 subjects (each with>90% bactericidal activity) were passed through a 50 kDa Centricon filter. Enriched activity was found in the flow through, although bactericidal activity was also found in the retentate, and silver stains of gels indicated that the separation of low and high molecular proteins was incomplete (not shown). Additional attempts to fractionate samples using the Superdex gel filtration column also yielded variable results. Therefore, we performed LC-MS/MS analysis on the unfractionated, flow-through and retentate (after passage through 50 kDA Centricon filter) from a CVL pool, 2 individual active samples (each with>90% bactericidal activity), and 2 inactive samples (<30% bactericidal activity), as well as a third individual active CVL and the most active fraction after separation in a gel filtration column.

There were 215 proteins identified in the CVL samples using thresholds of 80% protein probability, 95% peptide probability and one peptide per protein sequence from the Mascot searches, and each was validated using Scaffold 3.3 (Table S1). The protein and peptide false discovery rate are 2.0% and 0.1%, respectively. To identify proteins unique to the active samples, we used more stringent threshold values: protein identification probability of 95%, a minimum peptide probability of 95% and a minimum of 2 peptides per protein sequence. With these threshold values, the protein and peptide false discovery rate are 0%. The MS/MS raw data was also manually reviewed for peptide sequence confirmation resulting in the identification of 103 proteins. We eliminated those present in both active and inactive CVL, further reducing the results to 30 proteins, and then further narrowed the focus to those that were present in more than one active sample. After these adjustments, 6 unique proteins were detected in at least 2 active CVL samples (Table 3). These included 3 proteins from *L. crispatus* (S-layer protein, bacterial surface layer protein, and cell separation protein), 1 from *L. jensenii* (adhesion exoprotein) and 2 human proteins (bone-derived growth factor, and protein-glutamine γ- glutamyltransferase E precursor). The S-layer protein and bacterial surface protein, while not identical, share significant homology and may represent isoforms of the same protein. An example MS/MS spectrum of one peptide (DAILQLGGDALK) from *L. crispatus* S-layer protein is shown in Figure 3 where the matching sequence ions are clearly observed. A BLAST search against all species of this peptide matched to S-layer protein (ZP_03995927) and bacterial surface layer protein (ZP_06627035) of *L. crispatus* with matching e values of 0.011 (98% identical sequences).

### Spent Culture Supernatants from *L. jensenii* are Bactericidal for *E. coli*

To further explore the observation that lactobacillus proteins were identified in the active CVL, we examined the bactericidal activity of *L. jensenii* filtered SCS. The SCS were bactericidal for *E. coli*. Activity persisted over a pH range of 4.3–6.3, reflecting the pH of MRS and LB broth, respectively (Fig. 4), and was heat stable and resistant to proteinase K treatment (data not shown). In contrast, SCS from *G. vaginalis* exhibited little or no *E. coli* bactericidal activity (ongoing studies).

We then explored the possibility that women with greater bactericidal activity might be colonized with a greater proportion of lactobacillus species compared to women with reduced bactericidal activity. CVL pellets were available from 19 participants and were analyzed by PCR for concentrations of select species. The concentrations (copies DNA/CVL pellet) were variable (median [range]) 404.5 [50−2.5×10^8^] for *L. crispatus*; 50 [50−1.2×10^8^] for *L. jensenii*, 6.4×10^3^ [50−6.2×10^8^] for *L. iners* and 5.2×10^3^ [50−1.1×10^9^] for *G. vaginalis*, and did not correlate with bactericidal activity (47%, [−26–100]).

## Discussion

Results of this study confirm earlier observations that genital tract secretions can exhibit substantial bactericidal activity against *E. coli* [4] and suggest that proteins from commensal lactobacilli contribute to this activity. Lactobacilli species are the predominant microbiota of the vagina and contribute to innate host defense [12]–[14]. Prior studies focused on the antimicrobial activity of hydrogen peroxide, lactic acid, and bacteriocins as mediators of the antimicrobial activity of lactobacilli [15]–[17]. Bacteriocins are proteins produced by non-pathogenic bacteria that inhibit the growth of competing strains [18]. For example, in a recent study from Kenya, lactobacillus species were isolated in 82 of the 107 vaginal swabs collected, with *L. jensenii* predominating. Lactobacillus SCS were tested for antibacterial activity against BV-associated bacteria. Approximately 25% of the samples inhibited the growth of *Prevotella bivia*, *G. vaginalis* and *Mobiluncus* spp. Notably, the activity was lost if the samples were treated with proteinase K, suggesting that bacteriocins, which are proteinase K sensitive, contributed to the antimicrobial activity against the BV-associated bacteria [19].

In contrast, the CVL samples in the current study were only modestly and variably susceptible to proteinase K, suggesting that proteins distinct from bacteriocins may contribute to the *E. coli* bactericidal activity. Using proteomic approaches, we identified 4 lactobacillus proteins (3 originally described as proteins of *L. crispatus* and 1 of *L. jensenii*) present exclusively in active CVL and in more than one sample. Two of these, the S-layer protein and bacterial surface layer protein, which were initially identified in 2 different strains of *L. crispatus* (JV-V01 and CTV-05) and are not identical based on sequence data, may be isoforms or functionally similar proteins. The S-layer (or bacterial surface layer) proteins are components of the cell wall and are involved in bacterial adherence. Notably, SCS from a probiotic strain of lactobacillus, *L. kefir*, which contain significant amounts of S-layer protein, inhibit Salmonella invasion, implicating the S-layer protein in host defense [20]. Less is known about the function of the other lactobacilli proteins identified in the bactericidal CVL samples.

The importance of a healthy vaginal microbiome in preventing vaginal colonization with *E. coli* is illustrated by the observations that lactobacilli inhibit *E. coli* growth [21]. Proposed mechanisms include competitive exclusion [22], production of lactic acid, [4], and bacteriocins [23]. We extended these observations and found that the activity of *L. jensenii* SCS is pH independent, heat stable, and resistant to proteinase K, suggesting that additional proteins contribute to the bactericidal activity. Moreover, the bactericidal activity of SCS persists when lactic acid is inactivated by treatment with β-glycerophosphate (data not shown) further implicating other bacterial proteins as playing a role in defense against *E. coli.*

In a recent study, psoriasin (S100A7) co-eluted with a peak of *E. coli* killing activity in fractionated vaginal fluid samples, suggesting that this protein contributes to innate host defense [24]. We identified this protein in one active CVL sample; the protein was also potentially present in a second sample with a peptide identification probability of 88%.

Limitations of our proteomic analysis include the small number of samples evaluated, a focus on proteins exclusively identified in 2 or more active CVL, and the initial concentration of samples using sandwich gel electrophoresis with 10–12% acrylamide, which would not have retained smaller proteins (<10 kDa). By applying additional methods such as isobaric tag for relative and absolute quantification (iTRAQ), we may identify additional proteins that are present in different quantities in both active and inactive samples [25]. Confirmation of their role in mucosal defense will require depletion of candidate proteins from active CVL samples, assessment of the bactericidal activity of purified proteins, and reconstitution of activity in inactive CVL samples by adding back individual purified candidate proteins. Similarly, confirmation of the role of the lactobacillus proteins identified in the proteomic analysis will also require depletion experiments with bacterial SCS or organisms engineered without the candidate protein genes.

Another limitation is the paucity of samples available to evaluate the vaginal microbiota. We did not detect any association between bactericidal activity and concentrations of targeted Lactobacillus species. Unfortunately, vaginal swabs were not cultured to evaluate for *E. coli* colonization and CVL pellets were not available for *E. coli* qPCR. In a study with pregnant women, we found that *E. coli* bactericidal activity correlated inversely with *E. coli* colonization, supporting the contention that this activity may translate to clinical protection [26]. However, larger studies that include assessment of vaginal microbiota and bactericidal activity are needed.

Further studies are also needed to more precisely define the intersubject variability and to understand why CVL from 3 participants enhanced *E. coli* growth and what factors mediate this enhancing activity. Residual bacteria in the CVL supernatants seem unlikely as CVL from one subject inconsistently enhanced bacterial growth (5 of 7 isolates; Fig. 2C). Moreover, no bacteria were recovered by directly plating a random sampling of CVL supernatants.

In summary, the results of these studies suggest that both human and bacterial proteins may provide protection against *E. coli* colonization. Identification of the key mediators should facilitate the development of strategies to maintain this host defense and prevent colonization with pathogenic bacteria that increase the risk for UTIs, preterm labor and perinatal infection.

## Supporting Information

Table S1 **The 215 proteins identified in CVL samples using thresholds of 80% protein probability, 95% peptide probability and one peptide per protein sequence from the Mascot searches.** Each was validated using Scaffold 3.1 (Proteome Software, Portland, Oregon, USA). Sample 1 is a CVL pool of 5 active (>90% bactericidal activity) CVL, samples 2–4 are individual active samples, and samples 5 and 6 are individual CVL samples with<30% inhibitory activity.
(PDF)
