# Supplementary material for: Lactobacillus Proteins Are Associated with the Bactericidal Activity against E. coli of Female Genital Tract Secretions
Source: PLoS One. 2012 Nov 19;7(11):e49506. doi: 10.1371/journal.pone.0049506 (PMC3501525; doi:10.1371/journal.pone.0049506)
Supplement: Table S1 — The 215 proteins identified in CVL samples using thresholds of 80% protein probability, 95% peptide probability and one peptide per protein sequence from the Mascot searches. Each was validated using Scaffold 3.1 (Proteome Software, Portland, Oregon, USA). Sample 1 is a CVL pool of 5 active (>90% bactericidal activity) CVL, samples 2–4 are individual active samples, and samples 5 and 6 are individual CVL samples with<30% inhibitory activity. (PDF) [file pone.0049506.s001.pdf]

Table S1: The 215 proteins identified in CVL samples using thresholds of 80% protein probability, 95% peptide probability and one peptide per protein sequence from the Mascot searches. Each was validated using Scaffold 3.1 (Proteome Software, Portland, Oregon, USA). Sample 1 is a CVL pool of 5 active (>90% bactericidal activity) CVL, samples 2-4 are individual active samples, and samples 5 and 6 are individual CVL samples with < 30% inhibitory activity.

				Active Samples Protein probability (# of unique peptides)				Inactive Samples Protein probability (# of unique peptides	
#	Identified Proteins (215)	Accession Number	Molecular Weight (kDa)	1	2	3	4	5	6
1	Serum Albumin Complexed	122920512	66	100 (6)	100 (6)	100 (20)	100 (0)	100 (17)	100 (19)
2	Chain A, Trypsin In Complex With Borate	110590762	23	100 (2)	100 (2)	100 (3)	96 (0)	100 (4)	100 (2)
3	Transferrin	37747855	77	100 (5)	100 (2)	100 (24)	0 (0)	100 (18)	100 (21)
4	Lactotransferrin	18490850	78	100 (9)	100 (1)	100 (27)	99 (1)	100 (22)	0 (0)
5	keratin 1	11935049	66	100 (11)	100 (11)	100 (7)	100 (10)	100 (11)	100 (3)
6	squamous cell carcinoma antigen-1 isoform	193792580	45	100 (11)	100 (9)	100 (6)	91 (1)	100 (9)	100 (5)
7	Anti-TNF-alpha antibody light-chain Fab fragment	11275302	24	100 (7)	100 (2)	100 (8)	98 (1)	100 (5)	100 (7)
8	epidermal cytokeratin 2	181402	66	100 (6)	100 (6)	100 (5)	100 (7)	100 (8)	98 (1)
9	small proline-rich protein 3	4885607	18	100 (5)	100 (3)	100 (3)	0 (0)	100 (5)	100 (1)
10	Solution Structure Of Human Immunoglobulin M	166007160	57	100 (5)	100 (5)	100 (8)	49 (0)	100 (8)	100 (5)
11	Keratin 6C	84040267	60	100 (11)	100 (7)	100 (6)	100 (5)	100 (10)	0 (0)
12	Keratin 13	118835468	50	100 (10)	100 (4)	100 (4)	100 (2)	100 (11)	0 (0)
13	immunoglobin heavy constant alpha 1	34536000	53	100 (4)	100 (2)	100 (6)	0 (0)	100 (4)	100 (3)
14	Crystal Structure Of Myeloperoxidase	254220979	53	100 (12)	100 (0)	100 (14)	91 (1)	100 (6)	0 (0)
15	keratin 10	119581085	63	100 (7)	100 (5)	100 (2)	100 (8)	100 (4)	99 (1)
16	unnamed protein product, keratin	1335191	65	100 (14)	100 (3)	100 (10)	99 (1)	100 (13)	0 (0)
17	Annexin A2	16306978	39	100 (9)	100 (7)	100 (4)	54 (0)	100 (8)	0 (0)
18	S-layer protein [Lactobacillus crispatus JV-V01]	227877920	59	100 (10)	100 (8)	0 (0)	100 (1)	0 (0)	0 (0)
19	serum albumin	28590	69	98 (1)	82 (1)	100 (2)	0 (0)	100 (2)	100 (2)

20	alpha 2 macroglobulin variant	62088808	164	0 (0)	0 (0)	100 (10)	0 (0)	100 (4)	100 (17)
21	Solution Structure Of Human Secretory Component	146387599	64	100 (1)	100 (4)	100 (8)	0 (0)	100 (9)	0 (0)
22	alpha-2-macroglobulin-like protein 1 precursor	74271845	161	100 (5)	100 (6)	100 (5)	91 (1)	100 (5)	100 (9)
23	annexin A1, isoform CRA_b	119582950	40	100 (8)	100 (4)	100 (7)	0 (0)	100 (8)	100 (3)
24	leukocyte elastase inhibitor	13489087	43	100 (4)	100 (2)	100 (4)	0 (0)	100 (5)	83 (1)
25	mucin 5AC, oligomeric mucus/gel-forming	157057089	649	99 (1)	0 (0)	0 (0)	0 (0)	100 (18)	0 (0)
26	complement C3 precursor	115298678	187	87 (1)	0 (0)	100 (6)	0 (0)	100 (3)	100 (13)
27	alpha-1 antitrypsin variant	110350939	47	100 (1)	0 (0)	100 (2)	0 (0)	100 (3)	100 (3)
28	keratin, type II cytoskeletal 6A	5031839	60	100 (3)	100 (2)	100 (2)	100 (2)	100 (2)	0 (0)
29	Ig G1 H Nie	229601	49	100 (3)	100 (2)	100 (4)	0 (0)	100 (2)	100 (2)
30	IgGFc-binding protein precursor	154146262	572	98 (1)	100 (7)	100 (8)	0 (0)	100 (6)	0 (0)
31	immunoglobin heavy constant gamma 1	16554039	65	87 (1)	72 (0)	100 (2)	0 (0)	100 (1)	99 (1)
32	ceruloplasmin (ferroxidase), isoform CRA_b	119599289	123	58 (0)	0 (0)	100 (4)	0 (0)	100 (1)	100 (6)
33	keratin, type II cytoskeletal 5	119395754	62	100 (4)	100 (6)	100 (4)	100 (3)	100 (2)	0 (0)
34	SCCA-PD variant	27466917	45	87 (1)	82 (1)	81 (1)	0 (0)	79 (1)	83 (1)
35	Neutrophil Gelatinase-Associated Lipocalin (Hngal)	159162742	21	100 (3)	31 (0)	100 (2)	0 (0)	100 (3)	39 (0)
36	protein-glutamine gamma-glutamyltransferase	189458821	77	100 (3)	100 (4)	0 (0)	0 (0)	0 (0)	0 (0)
37	immunoglobulin kappa light chain	3169770	23	100 (3)	82 (1)	81 (1)	0 (0)	79 (1)	100 (3)
38	KRT9 protein	113197968	?	0 (0)	0 (0)	0 (0)	100 (9)	0 (0)	0 (0)
39	Immunoglobin heavy constant mu	34535866	52	87 (1)	82 (1)	100 (3)	0 (0)	100 (1)	100 (3)
40	Keratin 77	113414871	62	87 (1)	82 (1)	81 (1)	100 (1)	79 (1)	83 (1)
41	involucrin	386834	68	100 (1)	100 (2)	0 (0)	100 (1)	79 (1)	100 (3)
42	protein S100-A8	21614544	11	100 (2)	99 (1)	100 (2)	0 (0)	100 (2)	99 (1)
43	fatty acid-binding protein, epidermal	4557581	15	100 (1)	99 (1)	0 (0)	0 (0)	100 (1)	83 (1)
44	cytokeratin 9	435476	62	100 (1)	100 (3)	100 (2)	79 (0)	100 (2)	83 (1)
45	plastin-2	167614506	70	100 (2)	0 (0)	100 (6)	0 (0)	100 (1)	0 (0)
46	protein S100-A9	4506773	13	100 (2)	0 (0)	81 (1)	91 (1)	100 (2)	100 (1)
47	bacterial surface layer protein [Lactobacillus crispatus]	312983924	47	100 (3)	100 (2)	0 (0)	0 (0)	0 (0)	0 (0)
48	cornulin	7706635	54	100 (4)	100 (2)	100 (2)	91 (1)	100 (4)	100 (2)

49	immunoglobulin variable region	323433041	17	100 (2)	0 (0)	81 (1)	0 (0)	78 (1)	83 (1)
50	beta-actin	323472339	42	100 (3)	82 (1)	100 (3)	61 (0)	100 (2)	68 (0)
51	immunoglobulin lambda light chain C region	386813	15	87 (1)	82 (1)	81 (1)	0 (0)	79 (1)	83 (1)
52	immunoglobulin heavy chain constant alpha 1	169249224	36	87 (1)	82 (1)	81 (1)	0 (0)	79 (1)	83 (1)
53	JC-kappa protein - human	1082553	23	100 (2)	71 (0)	0 (0)	99 (0)	79 (1)	83 (1)
54	monoclonal IgM antibody heavy chain	41388180	64	0 (0)	0 (0)	100 (3)	0 (0)	100 (1)	100 (1)
55	adhesion exoprotein [Lactobacillus jensenii 208-1]	282932703	65	100 (2)	100 (3)	0 (0)	0 (0)	0 (0)	0 (0)
56	type II keratin subunit protein	386854	65	87 (1)	100 (2)	81 (1)	100 (2)	99 (1)	81 (0)
57	ly6/PLAUR domain-containing protein 3 precursor	93004088	36	99 (1)	100 (2)	81 (1)	0 (0)	79 (1)	83 (1)
58	glyceraldehyde-3-phosphate dehydrogenase[Lactobacillus crispatus]	227877217	37	100 (6)	61 (0)	0 (0)	0 (0)	79 (1)	0 (0)
59	immunoglobulin variable region	323432985	16	87 (1)	82 (1)	81 (1)	0 (0)	79 (1)	80 (0)
60	serum albumin	28592	69	0 (0)	0 (0)	100 (1)	0 (0)	79 (1)	100 (2)
61	immunoglobulin heavy chain constant region gamma 4	12054078	36	87 (1)	82 (1)	81 (1)	0 (0)	79 (1)	83 (1)
62	phosphopyruvate hydratase [Lactobacillus crispatus JV-V01]	227877402	47	100 (6)	0 (0)	0 (0)	0 (0)	0 (0)	0 (0)
63	alpha-1-B glycoprotein	119592981	54	0 (0)	0 (0)	100 (4)	0 (0)	100 (1)	100 (1)
64	B-cell receptor immunoglobulin heavy chain variable region	320117096	49	87 (1)	79 (0)	100 (2)	0 (0)	100 (0)	98 (0)
65	Ig kappa chain C region (allotype Inv(1,2)) - human (fragment)	106529	9	87 (1)	0 (0)	81 (1)	0 (0)	79 (1)	83 (1)
66	hemopexin precursor	11321561	52	0 (0)	0 (0)	99 (1)	0 (0)	100 (2)	100 (1)
67	PRO2044	6650826	68	87 (1)	82 (1)	81 (1)	0 (0)	35 (0)	0 (0)
68	Chain A, Refined Solution Structure Of Human Cystatin A	15988456	11	98 (1)	100 (1)	0 (0)	0 (0)	0 (0)	100 (1)
69	Transferrin	194383506	75	0 (0)	0 (0)	100 (3)	0 (0)	79 (1)	100 (1)
70	immunoglobulin heavy chain constant region	10799664	36	28 (0)	0 (0)	81 (1)	0 (0)	57 (0)	83 (1)
71	pyruvate kinase [Lactobacillus crispatus JV-V01]	227879085	63	100 (5)	0 (0)	0 (0)	0 (0)	0 (0)	0 (0)
72	carcinoembryonic antigen precursor	178677	77	100 (2)	100 (3)	0 (0)	0 (0)	79 (1)	0 (0)
73	elongation factor Tu [Lactobacillus crispatus JV-V01]	227877363	44	100 (5)	0 (0)	0 (0)	0 (0)	0 (0)	0 (0)
74	Collagen, type I, alpha 2	32451581	129	0 (0)	0 (0)	0 (0)	0 (0)	100 (5)	0 (0)
75	immunoglobulin lambda 2 light chain	170684534	23	87 (1)	0 (0)	46 (0)	85 (0)	79 (1)	83 (1)

76	immunoglobulin light chain	149673887	23	87 (1)	0 (0)	81 (1)	0 (0)	79 (1)	83 (1)
77	proapolipoprotein	178775	29	0 (0)	0 (0)	0 (0)	0 (0)	0 (0)	100 (6)
78	Keratin 14	12803709	52	90 (0)	100 (0)	69 (0)	99 (1)	44 (0)	0 (0)
79	polymeric immunoglobulin receptor precursor	31377806	83	0 (0)	82 (1)	81 (1)	0 (0)	79 (1)	0 (0)
80	Chain A, Apo-Human Serum Transferrin (Non-Glycosylated)	110590597	75	0 (0)	0 (0)	100 (1)	0 (0)	0 (0)	100 (1)
81	keratin type II cytoskeletal 4	82654947	58	98 (1)	75 (0)	81 (1)	99 (1)	48 (0)	0 (0)
82	immunoglobulin kappa 4 light chain	170684576	24	87 (1)	0 (0)	81 (1)	0 (0)	79 (1)	83 (1)
83	medullasin	219923	25	100 (2)	0 (0)	100 (1)	0 (0)	100 (2)	0 (0)
84	annexin A3, isoform CRA_b	119626228	40	100 (2)	0 (0)	100 (2)	0 (0)	79 (1)	0 (0)
85	LgsC [Lactobacillus crispatus MV-1A-US]	256849292	53	99 (1)	100 (1)	0 (0)	0 (0)	0 (0)	0 (0)
86	suprabasin isoform 1 precursor	260436922	61	100 (2)	82 (1)	81 (1)	86 (0)	79 (1)	0 (0)
87	desmoplakin I	1147813	332	100 (2)	36 (0)	81 (1)	82 (0)	100 (2)	0 (0)
88	small proline rich protein	338421	10	87 (1)	82 (1)	0 (0)	0 (0)	79 (1)	0 (0)
89	heat shock protein 27	662841	22	100 (1)	35 (0)	0 (0)	0 (0)	100 (2)	0 (0)
90	Chain A, Human Cathepsin G	3891975	25	100 (1)	0 (0)	98 (0)	0 (0)	0 (0)	0 (0)
91	complement component C4A	179674	193	0 (0)	0 (0)	81 (1)	0 (0)	0 (0)	100 (3)
92	keratin 5b, isoform CRA_a	119617055	45	100 (2)	75 (0)	81 (1)	91 (1)	0 (0)	0 (0)
93	keratin 3, isoform CRA_b	119617052	57	100 (2)	100 (1)	81 (1)	0 (0)	0 (0)	0 (0)
94	92 kDa type IV collagenase	177205	78	100 (2)	0 (0)	81 (1)	0 (0)	79 (1)	0 (0)
95	SNF5/INI1 protein	3326993	41	0 (0)	0 (0)	0 (0)	0 (0)	100 (3)	0 (0)
96	keratin 4	119617053	65	87 (1)	75 (0)	81 (1)	91 (1)	79 (1)	0 (0)
97	beta-actin	89276723	42	87 (1)	0 (0)	81 (1)	0 (0)	100 (2)	100 (2)
98	alpha-enolase isoform 1	4503571	47	83 (0)	0 (0)	100 (0)	0 (0)	79 (1)	100 (2)
99	immunoglobulin light chain	149673889	23	98 (1)	0 (0)	81 (1)	0 (0)	79 (1)	83 (1)
100	immunoglobulin heavy chain	10334587	41	0 (0)	0 (0)	99 (1)	0 (0)	76 (0)	100 (1)
101	hCG22067	119572363	30	57 (0)	82 (1)	0 (0)	39 (0)	41 (0)	31 (0)
102	bone-derived growth factor	1203965	86	98 (1)	100 (3)	0 (0)	0 (0)	0 (0)	0 (0)
103	hornerin precursor	40795897	282	0 (0)	98 (1)	0 (0)	100 (3)	0 (0)	0 (0)
104	complement factor H	77744385	139	0 (0)	0 (0)	0 (0)	0 (0)	0 (0)	100 (6)

105	serpin peptidase inhibitor, clade B (ovalbumin)	119583554	40	87 (1)	82 (1)	0 (0)	91 (1)	79 (1)	0 (0)
106	type I keratin 16	1195531	51	0 (0)	63 (0)	69 (0)	99 (1)	100 (2)	0 (0)
107	poly-Ig receptor, partial	514366	83	0 (0)	54 (0)	81 (1)	0 (0)	79 (1)	0 (0)
108	haptoglobin, isoform CRA_a	119579598	47	0 (0)	0 (0)	55 (0)	0 (0)	21 (0)	100 (2)
109	azurocidin	28977	27	87 (1)	0 (0)	0 (0)	0 (0)	0 (0)	0 (0)
110	immunoglobulin mu heavy chain	54778900	25	87 (1)	82 (1)	81 (1)	0 (0)	79 (1)	83 (1)
111	Ig heavy chain variable region, VH3 family	33318934	13	87 (1)	82 (1)	81 (1)	0 (0)	0 (0)	83 (1)
112	immunoglobulin light chain variable region	8176546	11	87 (1)	0 (0)	81 (1)	0 (0)	79 (1)	0 (0)
113	cornifin-A	45827734	10	0 (0)	82 (1)	81 (1)	0 (0)	79 (1)	0 (0)
114	myosin-9	12667788	227	46 (0)	0 (0)	81 (1)	0 (0)	100 (3)	0 (0)
115	cell separation protein [Lactobacillus crispatus 125-2-CHN]	256844356	56	100 (2)	100 (1)	0 (0)	0 (0)	0 (0)	0 (0)
116	histone H2A - human	2118981	24	85 (0)	99 (1)	0 (0)	0 (0)	0 (0)	0 (0)
117	kallikrein 13 precursor	197692631	31	87 (1)	0 (0)	0 (0)	0 (0)	79 (1)	0 (0)
118	maltose ATP binding cassette transporter [Lactobacillus crispatus]	227878509	44	100 (3)	59 (0)	0 (0)	0 (0)	0 (0)	0 (0)
119	phosphoglycerate kinase [Lactobacillus crispatus JV-V01]	227877218	46	100 (2)	0 (0)	0 (0)	0 (0)	0 (0)	0 (0)
120	immunoglobulin heavy chain variable region	118405839	13	100 (2)	82 (1)	0 (0)	0 (0)	79 (1)	83 (1)
121	aldolase A, fructose-bisphosphate, isoform CRA_b	119600342	40	0 (0)	96 (0)	81 (1)	0 (0)	0 (0)	100 (2)
122	immunoglobulin gamma heavy chain	316996524	24	87 (1)	0 (0)	99 (1)	0 (0)	0 (0)	79 (0)
123	myeloperoxidase	2160397	81	87 (1)	0 (0)	79 (0)	0 (0)	43 (0)	0 (0)
124	Ig alpha-2 chain C region (allotype A2m(2)) - human (fragment)	87783	24	87 (1)	82 (1)	0 (0)	0 (0)	0 (0)	0 (0)
125	hemoglobin beta chain	66731527	11	0 (0)	0 (0)	0 (0)	0 (0)	0 (0)	100 (2)
126	Vinculin	24657579	117	0 (0)	100 (1)	0 (0)	0 (0)	0 (0)	0 (0)
127	keratin, type I cytoskeletal 15	24430190	49	87 (1)	99 (1)	69 (0)	78 (0)	0 (0)	0 (0)
128	serpin peptidase inhibitor	119601994	51	87 (1)	0 (0)	81 (1)	0 (0)	56 (0)	100 (2)
129	Plakoglobin	194373749	63	87 (1)	63 (0)	69 (0)	78 (0)	0 (0)	0 (0)
130	bactericidal permeability-increasing protein precursor	157276599	54	100 (2)	0 (0)	81 (1)	0 (0)	79 (1)	0 (0)
131	keratin, type II cytoskeletal 79	32567786	65	100 (2)	75 (0)	81 (1)	0 (0)	0 (0)	0 (0)
132	immunoglobulin kappa light chain VLJ region	21669331	29	87 (1)	0 (0)	81 (1)	0 (0)	0 (0)	75 (0)

133	psoriasin	12053626	10	99 (1)	95 (0)	0 (0)	0 (0)	0 (0)	0 (0)
134	immunoglobulin gamma heavy chain	327553627	17	0 (0)	0 (0)	81 (1)	0 (0)	38 (0)	0 (0)
135	gelsolin isoform c	189083780	82	0 (0)	0 (0)	0 (0)	0 (0)	0 (0)	100 (4)
136	Ig heavy chain V region (DP-30) - human (fragment)	284187	51	0 (0)	0 (0)	0 (0)	0 (0)	100 (3)	0 (0)
137	Molecular Envelope Structure Of Type I Collagen	254221096	93	0 (0)	0 (0)	0 (0)	0 (0)	100 (2)	0 (0)
138	immunoglobulin kappa 2 light chain	156633170	13	87 (1)	0 (0)	0 (0)	91 (1)	79 (1)	0 (0)
139	heat shock 70 kDa protein 1A/1B	167466173	70	98 (1)	0 (0)	81 (1)	0 (0)	0 (0)	74 (0)
140	immunoglobulin heavy chain variable region	27753271	13	87 (1)	0 (0)	100 (2)	0 (0)	0 (0)	30 (0)
141	immunoglobulin light chain	194173393	23	87 (1)	0 (0)	0 (0)	0 (0)	0 (0)	83 (1)
142	non-specific cross reacting antigen	189085	37	87 (1)	82 (1)	0 (0)	0 (0)	0 (0)	0 (0)
143	immunoglobulin light chain variable region	4323183	12	87 (1)	0 (0)	0 (0)	0 (0)	79 (1)	0 (0)
144	deleted in malignant brain tumors 1	55962152	274	87 (1)	0 (0)	0 (0)	0 (0)	79 (1)	0 (0)
145	immunoglobulin mu heavy chain	54779329	24	0 (0)	0 (0)	81 (1)	0 (0)	79 (1)	0 (0)
146	vitamin D-binding protein/group specific component	455970	53	0 (0)	0 (0)	0 (0)	0 (0)	0 (0)	100 (3)
147	keratin type II	386849	60	87 (1)	0 (0)	0 (0)	91 (1)	79 (1)	0 (0)
148	Enolase 1, (alpha) [Danio rerio]	37590349	47	87 (1)	0 (0)	79 (0)	0 (0)	0 (0)	83 (1)
149	Periplakin	109731912	205	100 (2)	0 (0)	0 (0)	0 (0)	97 (0)	0 (0)
150	alpha-2-macroglobulin precursor	66932947	164	0 (0)	0 (0)	81 (1)	0 (0)	0 (0)	100 (2)
151	alpha-2-macroglobulin, partial	177872	164	0 (0)	0 (0)	81 (1)	0 (0)	0 (0)	83 (1)
152	40S ribosomal protein S16	4506691	16	87 (1)	77 (0)	0 (0)	0 (0)	0 (0)	0 (0)
153	lectin, galactoside-binding, soluble, 3 binding protein	119609949	65	0 (0)	0 (0)	0 (0)	0 (0)	100 (2)	0 (0)
154	glutathione S-transferase	2204207	23	99 (1)	0 (0)	0 (0)	0 (0)	0 (0)	0 (0)
155	pyruvate kinase [Lactobacillus crispatus ST1]	295692870	63	87 (1)	0 (0)	0 (0)	0 (0)	0 (0)	0 (0)
156	lactoferrin	193527456	78	87 (1)	0 (0)	98 (1)	0 (0)	0 (0)	0 (0)
157	immunoglobulin kappa light chain variable region	98956266	23	87 (1)	0 (0)	0 (0)	0 (0)	0 (0)	83 (1)
158	fibrinogen alpha chain isoform alpha-E preproprotein	4503689	95	87 (1)	0 (0)	0 (0)	0 (0)	0 (0)	83 (1)
159	Ig heavy chain V-III region (ART) - human (fragments)	106482	24	87 (1)	0 (0)	81 (1)	0 (0)	0 (0)	0 (0)
160	aldolase A protein	28595	39	0 (0)	0 (0)	81 (1)	0 (0)	0 (0)	83 (1)
161	fructose-bisphosphate aldolase class-II [Lactobacillus iners]	259501426	33	100 (2)	0 (0)	0 (0)	0 (0)	0 (0)	0 (0)

162	fibrinogen beta chain, isoform CRA_d	119625338	52	0 (0)	0 (0)	0 (0)	0 (0)	0 (0)	100 (2)
163	Complement factor B	13278732	86	0 (0)	0 (0)	0 (0)	0 (0)	0 (0)	100 (1)
164	flavin-nucleotide-binding protein [Lactobacillus crispatus JV-V01]	227878595	14	100 (2)	0 (0)	0 (0)	0 (0)	0 (0)	0 (0)
165	alpha-1-microglobulin	579676	17	0 (0)	0 (0)	0 (0)	0 (0)	0 (0)	100 (2)
166	Chromosome 20 open reading frame 114	14250058	52	0 (0)	0 (0)	99 (1)	0 (0)	0 (0)	0 (0)
167	immunoglobulin lambda chain variable region	587404	13	87 (1)	0 (0)	0 (0)	0 (0)	0 (0)	0 (0)
168	antithrombin III	179161	53	0 (0)	0 (0)	0 (0)	0 (0)	0 (0)	83 (1)
169	trypsin inhibitor	33985	107	0 (0)	0 (0)	0 (0)	0 (0)	0 (0)	83 (1)
170	Ig kappa chain precursor V-II region - human (fragment)	418845	12	0 (0)	0 (0)	81 (1)	0 (0)	0 (0)	83 (1)
171	serine protease inhibitor Kazal type 5	9027546	121	83 (0)	82 (1)	0 (0)	0 (0)	0 (0)	0 (0)
172	immunoglobulin kappa light chain variable region	215982746	13	87 (1)	0 (0)	0 (0)	0 (0)	46 (0)	0 (0)
173	Ig alpha-2 chain C region - human	70058	37	87 (1)	0 (0)	39 (0)	0 (0)	0 (0)	0 (0)
174	ATP synthase subunit beta [Clostridium paradoxum]	77964181	50	99 (1)	0 (0)	0 (0)	0 (0)	0 (0)	0 (0)
175	cystatin-B	4503117	11	98 (1)	0 (0)	0 (0)	0 (0)	0 (0)	0 (0)
176	keratin 10, partial	186629	47	87 (1)	0 (0)	0 (0)	0 (0)	0 (0)	0 (0)
177	NADH peroxidase [Lactobacillus crispatus JV-V01]	227876994	49	87 (1)	0 (0)	0 (0)	0 (0)	0 (0)	0 (0)
178	chaperonin GroEL [Lactobacillus crispatus JV-V01]	227877610	58	87 (1)	0 (0)	0 (0)	0 (0)	0 (0)	0 (0)
179	D-lactate dehydrogenase [Lactobacillus crispatus JV-V01]	227878043	38	87 (1)	0 (0)	0 (0)	0 (0)	0 (0)	0 (0)
180	transglutaminase K enzyme	339604	89	87 (1)	0 (0)	0 (0)	0 (0)	0 (0)	0 (0)
181	alpha-tubulin	37492	50	87 (1)	0 (0)	0 (0)	0 (0)	0 (0)	0 (0)
182	Phospholipase B domain containing 1	39645313	63	0 (0)	82 (1)	0 (0)	0 (0)	0 (0)	0 (0)
183	PRSS3 protein	118763987	26	0 (0)	0 (0)	0 (0)	91 (1)	0 (0)	0 (0)
184	galectin-7	4504985	15	0 (0)	0 (0)	0 (0)	91 (1)	0 (0)	0 (0)
185	Ig kappa chain V-III region (Kas) - human (fragment)	106601	12	87 (1)	0 (0)	0 (0)	0 (0)	0 (0)	0 (0)
186	ERO1-like (S. cerevisiae), isoform CRA_b	119586051	54	87 (1)	0 (0)	0 (0)	0 (0)	0 (0)	0 (0)
187	12-lipoxygenase	189774	76	87 (1)	0 (0)	0 (0)	0 (0)	0 (0)	0 (0)
188	immunoglobulin light chain variable region	340764286	23	87 (1)	0 (0)	0 (0)	0 (0)	0 (0)	0 (0)
189	kallikrein-10 preproprotein	22208982	30	87 (1)	0 (0)	0 (0)	0 (0)	0 (0)	0 (0)
190	ATP binding cassette transporter protein	227878508	41	87 (1)	0 (0)	0 (0)	0 (0)	0 (0)	0 (0)

	[Lactobacillus crispatus]								
191	30S ribosomal protein S1 [Lactobacillus crispatus JV-V01]	227879095	44	87 (1)	0 (0)	0 (0)	0 (0)	0 (0)	0 (0)
192	maltose ABC transporter [Lactobacillus jensenii]	282934293	43	87 (1)	0 (0)	0 (0)	0 (0)	0 (0)	0 (0)
193	glutamyl-tRNA synthetase [Lactobacillus crispatus 214-1]	293381072	58	87 (1)	0 (0)	0 (0)	0 (0)	0 (0)	0 (0)
194	glutamine synthetase, type I [Lactobacillus crispatus CTV-05]	312977118	49	87 (1)	0 (0)	0 (0)	0 (0)	0 (0)	0 (0)
195	basic membrane protein [Lactobacillus crispatus CTV-05]	312984070	39	87 (1)	0 (0)	0 (0)	0 (0)	0 (0)	0 (0)
196	gamma-glutamylcyclotransferase isoform 4	315360622	11	87 (1)	0 (0)	0 (0)	0 (0)	0 (0)	0 (0)
197	immunoglobulin kappa light chain	33235614	12	87 (1)	0 (0)	0 (0)	0 (0)	0 (0)	0 (0)
198	SCCA2/SCCA1 fusion protein isoform 1	33317676	45	87 (1)	0 (0)	0 (0)	0 (0)	0 (0)	0 (0)
199	Ig heavy chain variable region, VH3 family	33319012	13	87 (1)	0 (0)	0 (0)	0 (0)	0 (0)	0 (0)
200	40S ribosomal protein S14	5032051	16	87 (1)	0 (0)	0 (0)	0 (0)	0 (0)	0 (0)
201	HSP90AA1 protein	83318444	68	87 (1)	0 (0)	0 (0)	0 (0)	0 (0)	0 (0)
202	tyrosine 3-monooxygenasea	86651742	12	87 (1)	0 (0)	0 (0)	0 (0)	0 (0)	0 (0)
203	Fibronectin 1	109658664	240	0 (0)	0 (0)	0 (0)	0 (0)	0 (0)	83 (1)
204	transthyretin	114318993	20	0 (0)	0 (0)	0 (0)	0 (0)	0 (0)	83 (1)
205	Hemoglobin subunit beta	122640	16	0 (0)	0 (0)	0 (0)	0 (0)	0 (0)	83 (1)
206	serine protease inhibitor Kazal-type 7 precursor	14211875	9	0 (0)	0 (0)	0 (0)	0 (0)	0 (0)	83 (1)
207	glyceraldehyde-3-phosphate dehydrogenase	182979	36	0 (0)	0 (0)	0 (0)	0 (0)	0 (0)	83 (1)
208	Histidine-rich glycoprotein	32454	4	0 (0)	0 (0)	0 (0)	0 (0)	0 (0)	83 (1)
209	afamin precursor	4501987	69	0 (0)	0 (0)	0 (0)	0 (0)	0 (0)	83 (1)
210	immunoglobulin lambda light chain	6467839	23	0 (0)	0 (0)	0 (0)	0 (0)	0 (0)	83 (1)
211	protein S100-A11	5032057	12	0 (0)	0 (0)	0 (0)	0 (0)	0 (0)	83 (1)
212	peroxiredoxin 1	55959887	19	0 (0)	0 (0)	0 (0)	0 (0)	0 (0)	82 (1)
213	Ribosomal protein L14	12653649	24	0 (0)	82 (1)	0 (0)	0 (0)	0 (0)	0 (0)
214	plakophilin-1 isoform 1b	53729346	83	0 (0)	82 (1)	0 (0)	0 (0)	0 (0)	0 (0)
215	cytochrome b-245 heavy chain	6996021	65	0 (0)	0 (0)	81 (1)	0 (0)	0 (0)	0 (0)
